# Synthesis, non-spherical structure refinement and Hirshfeld surface analysis of racemic 2,2′-diisobut­oxy-1,1′-bi­naphthalene

**DOI:** 10.1107/S2056989024009101

**Published:** 2024-09-24

**Authors:** Pénayori Marie-Aimée Coulibaly, Eric Ziki, Yvon Bibila Mayaya Bisseyou, Tchambaga Etienne Camara, Souleymane Coulibaly, Drissa Sissouma

**Affiliations:** a Laboratoire de Constitution et de Réaction de la Matière, Equipe Synthèse Organique, UFR de Sciences des Structures de la Matière et Technologie, Université, Félix Houphouët Boigny, 22 BP 582 Abidjan 22, Côte d’Ivoire; b Laboratoire des Sciences de la Matière,de l’Environnement et de l’Energie Solaire, Equipe de Recherche de Cristallographie et Physique Moléculaire, Université Félix Houphouët-Boigny, 08 BP 582, Abidjan 22, Côte d’Ivoire; University of Aberdeen, United Kingdom

**Keywords:** 1–1′-binaphthyl derivative, non-spherical refinement, Hirshfeld surface analysis, crystal structure

## Abstract

In the racemic title compound, C_28_H_30_O_2_, the naphthyl ring systems subtend a dihedral angle of 68.59 (1)° and the mol­ecular conformation is consolidated by a pair of intra­molecular C—H⋯π contacts. The crystal packing features a weak C—H⋯π contact and van der Waals forces. A Hirshfeld surface analysis of the crystal structure reveals that the most significant contributions are from H⋯H (73.2%) and C⋯H/H⋯C (21.2%) contacts.

## Abstract 2

The structure of the title 1–1′-binaphthyl derivative was refined with non-spherical atomic form factors computed using the DFT method at the PBE0/def2-TZVP basis set level.

## 1. Chemical context

1-1′-Binaphthyl-based systems play an important role as ligands in the design of optically active materials (Tkachenko & Scheiner, 2019 ▸). They are also used for mol­ecular recog­nition and asymmetric catalysis (Pu, 1998 ▸, 2024 ▸). The non-coplanar conformation of both naphthyl moieties coupled to their restricted rotation around the transannular covalent bond are the basis of their optical activity (chirality). Furthermore, it has been shown that, when substitutions are carried out at the 2,2′-positions of the 1-1′-binaphthyl system, the chiral conformation of the resulting derivative is very stable (Hall & Turner, 1955 ▸; Dixon *et al.*, 1971 ▸). Among these, 1-1′-bi-2-naphthol, C_20_H_14_O_2_, known as BINOL, with local *C*_2_ symmetry, has been used extensively in the production of chiral catalysts, dendrimers, mol­ecular probes, metal–organic frameworks, covalent organic frameworks, *etc*. In addition, its hydroxyl functions can be functionalized to generate a wide range of 1-1′-binaphthyl derivatives. Many synthesis procedures of racemic BINOL have been developed using oxidizing agents such as Fe^3+^, Cu^2+^, Mn^3+^, Ti^2+^, Co^3+^, Ag^2+^ or Mo^5+^ (Waldvogel, 2002 ▸; Doussot *et al.*, 2000 ▸; McKillop *et al.*, 1980 ▸; Budniak *et al.*, 2017 ▸) and pure enanti­omers can be obtained from the their racemates *via* diastereoisomer derivatives and their resolution can be achieved by the formation of inclusion crystals with chiral host mol­ecules (Hara *et al.*, 2002 ▸). Another strategy involves the deracemization of racemates with copper complexes of chiral amines (Bringmann *et al.*, 1990 ▸; Smrcina *et al.*, 1992 ▸) or by enzymatic hydrolysis of esters (Miyamo *et al.*, 1987 ▸; Kazlauskas *et al.*, 1989 ▸). Optically pure enanti­omers can also be synthesized directly and several methods have been reported for this purpose (Chow *et al.*, 1996 ▸; Kawashima & Hirata, 1993 ▸; Wang *et al.*, 1995 ▸).
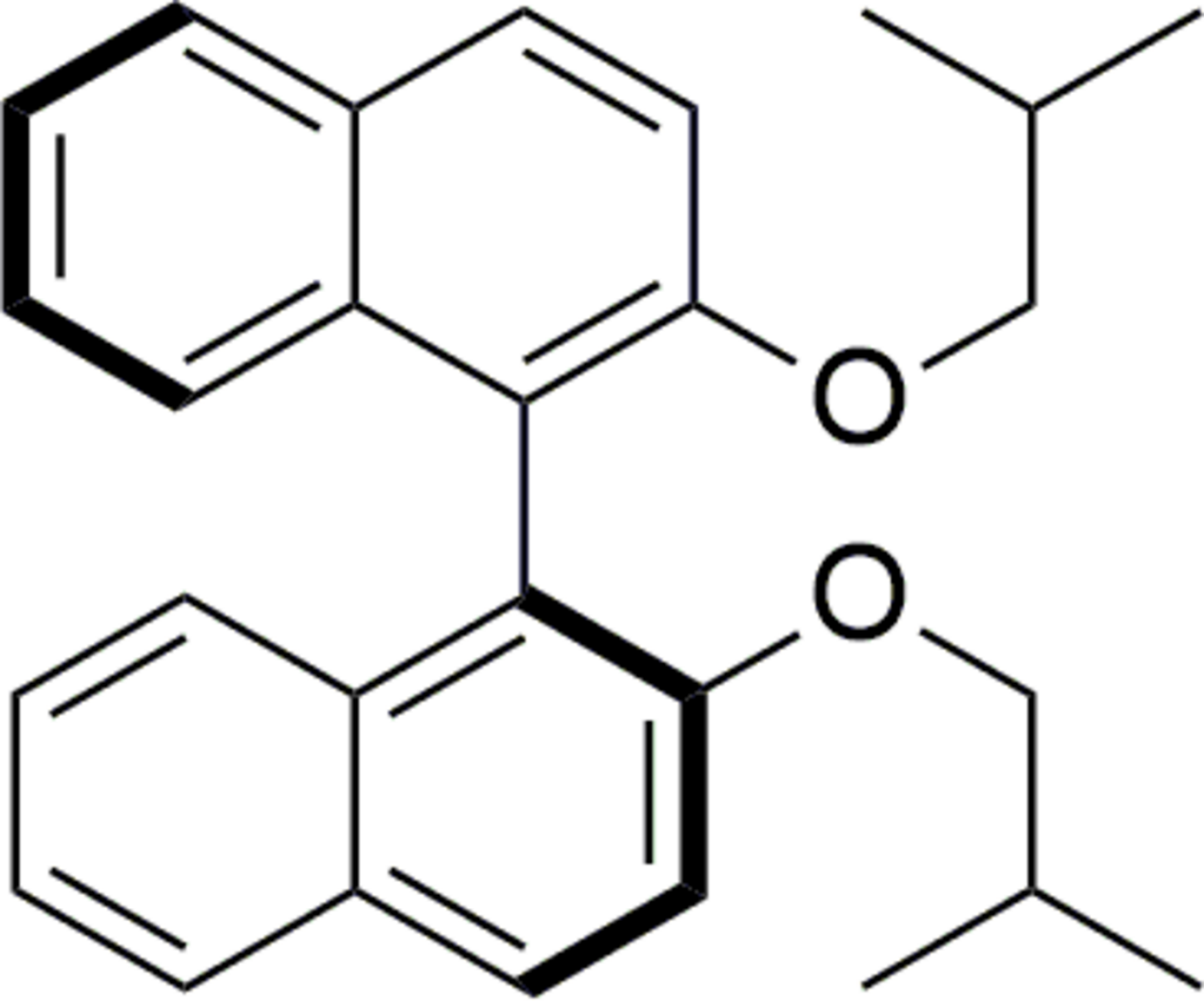


The racemic title compound, C_28_H_30_O_2_ (I), is a 1-1′-bi­naphthyl derivative obtained by the functionalization of BINOL at the hydroxyl positions. Herein, we report its synthesis, spectroscopic characterization and mol­ecular geometry, determined from single-crystal X-ray diffraction analysis using a non-standard aspherical refinement, which combines quantum mechanical calculations and data from diffraction experiments into a single integrated tool (Jayatilaka & Dittrich, 2008 ▸; Capelli *et al.*, 2014 ▸; Kleemiss *et al.*, 2021 ▸). A Hirshfeld surface analysis (Spackman & Byrom, 1997 ▸; McKinnon *et al.*, 1998 ▸; McKinnon *et al.*, 2004 ▸) of the title compound was also performed.

## 2. Structural commentary

**Figure 1:**
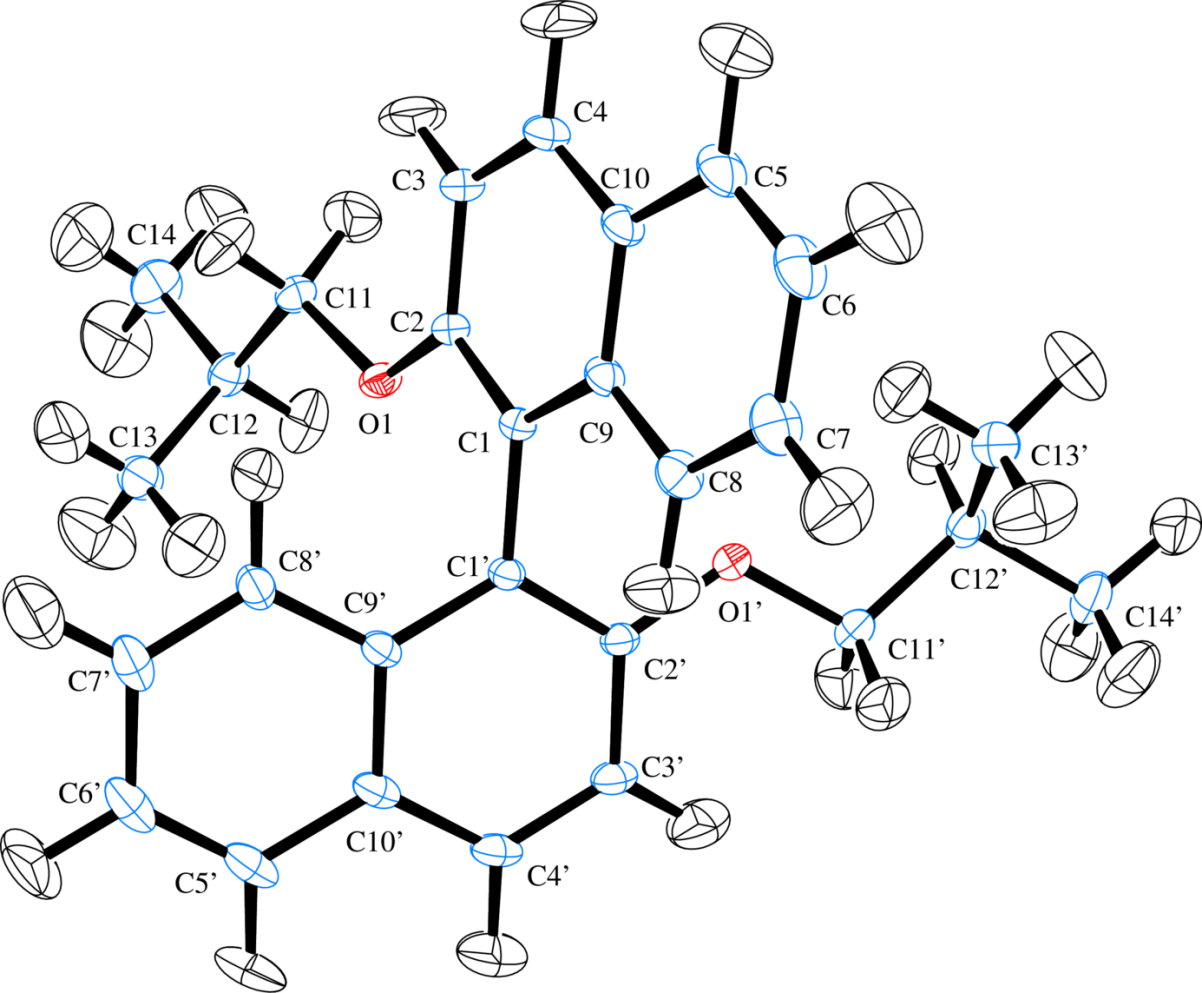
The mol­ecular structure of (I) with displacement ellipsoids for all atoms including H drawn at the 50% probability level.

**Figure 2:**
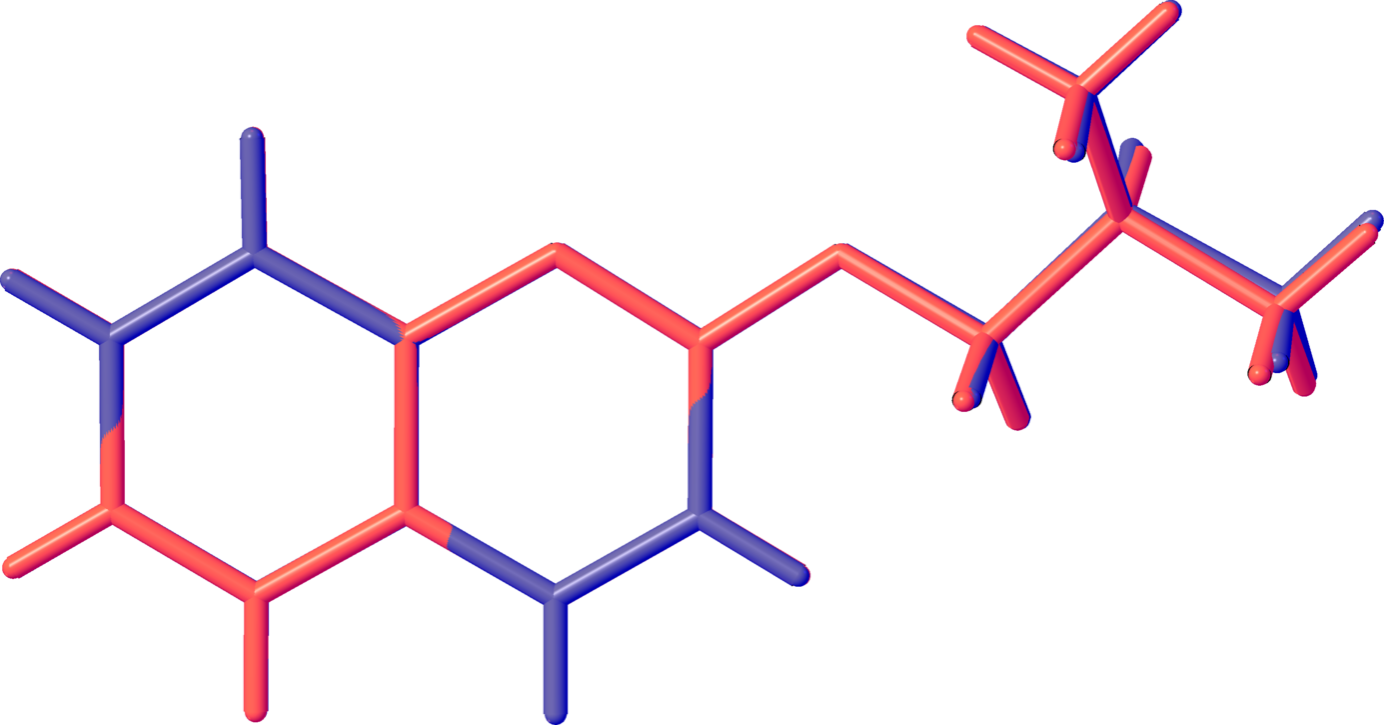
An overlay diagram of the β-naphthyl isobutyl ether moieties of (I).

**Figure 3:**
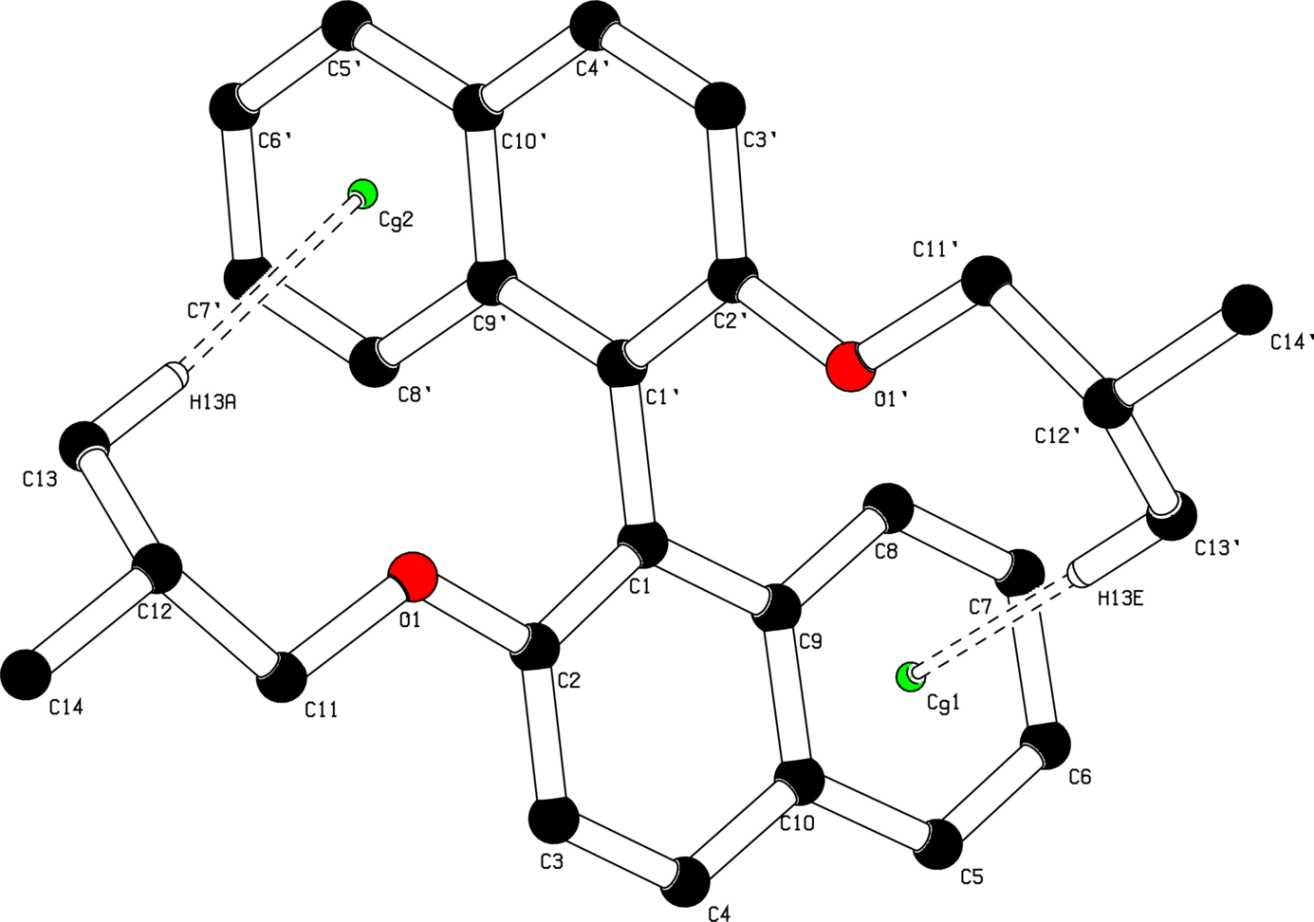
The intra­molecular C—H⋯π inter­actions in (I), shown as double dotted lines with centroids as green spheres.

Compound (I) crystallizes in space group *P*2_1_/*c* as a racemate (Fig. 1 ▸). The mol­ecular structure comprises two β-naphthyl isobutyl ether moieties linked by a C1—C1′ covalent bond whose length [1.4867 (3) Å] is in good agreement with the values reported for the same type of bond in related compounds (Allen & Bruno, 2010 ▸). Both ether units adopt extended conformations [C2—O1—C11—C12 = −175.59 (2)°; C2′—O1′—C11′—C12′ = −175.12 (2)°] and the C_14_H_15_O β-naphthyl isobutyl ether moieties exhibit very similar structural parameters with an alignment r.m.s.d. value of 0.023 Å (Fig. 2 ▸). The aromatic C—C bonds of the naphthyl ring systems have values in the same range as those obtained by Rivera *et al.* (2017 ▸). The planes of the naphthyl ring systems C1–C10 and C1′–C10′ (r.m.s deviations of 0.013 Å and 0.037 Å, respectively) form a twist angle of 68.59 (1)° compared to 68.52 (5)° in BINOL (ref, date). The C_ar_—O and C_alk­yl_—O ether bond lengths in (I) have comparable values to those found in related structures (Allen & Bruno, 2010 ▸). The mol­ecular conformation of (I) is consolidated (Table 1 ▸) by intra­molecular C13′—H13*E*⋯*Cg*1 and C13—H13*A*⋯*Cg*2 contacts, where *Cg*1 and *Cg*2 are the centroids of the C5–C10 and C5′–C10′ rings, respectively (Fig. 3 ▸, Table 1 ▸).

## 3. Supra­molecular features

Although the mol­ecule has two potential hydrogen-bond acceptor sites (atoms O1 and O2), no hydrogen bonding was found in this crystal structure. Indeed, the minimization of steric effects within the mol­ecule gives rise to a structural geometry whose intrinsic mol­ecular and environmental parameters in the crystal would prevent the formation of hydrogen bonding. However, analysis using *PLATON* (Spek, 2020 ▸) reveals a C13—H13*C*⋯*Cg*2(*x*, 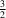 − *y*, 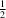 + *z*) (Table 1 ▸). In addition, the packing displays several C—H⋯H—C inter­molecular contacts ranging from 2.33 to 2.53 Å. Matta (2006 ▸) reported that these closed-shell inter­molecular inter­actions exhibit energetic stability, potentials typical of bound systems and stable equilibrium geometries.

## 4. Database survey

A search of the Cambridge Structural Database (CSD version 5.45; Groom *et al.*, 2016 ▸) for compounds containing the 1-1′-binaphthyl system with an ether moiety linked at the 2,2′ positions gave two hits [CSD refcodes PONTAO (Thoss *et al.*, 2009 ▸) and PONTAO01 (Maria *et al.*, 2017 ▸)], in which the asymmetric units contain two mol­ecules.

## 5. Hirshfeld surface analysis

**Figure 4:**
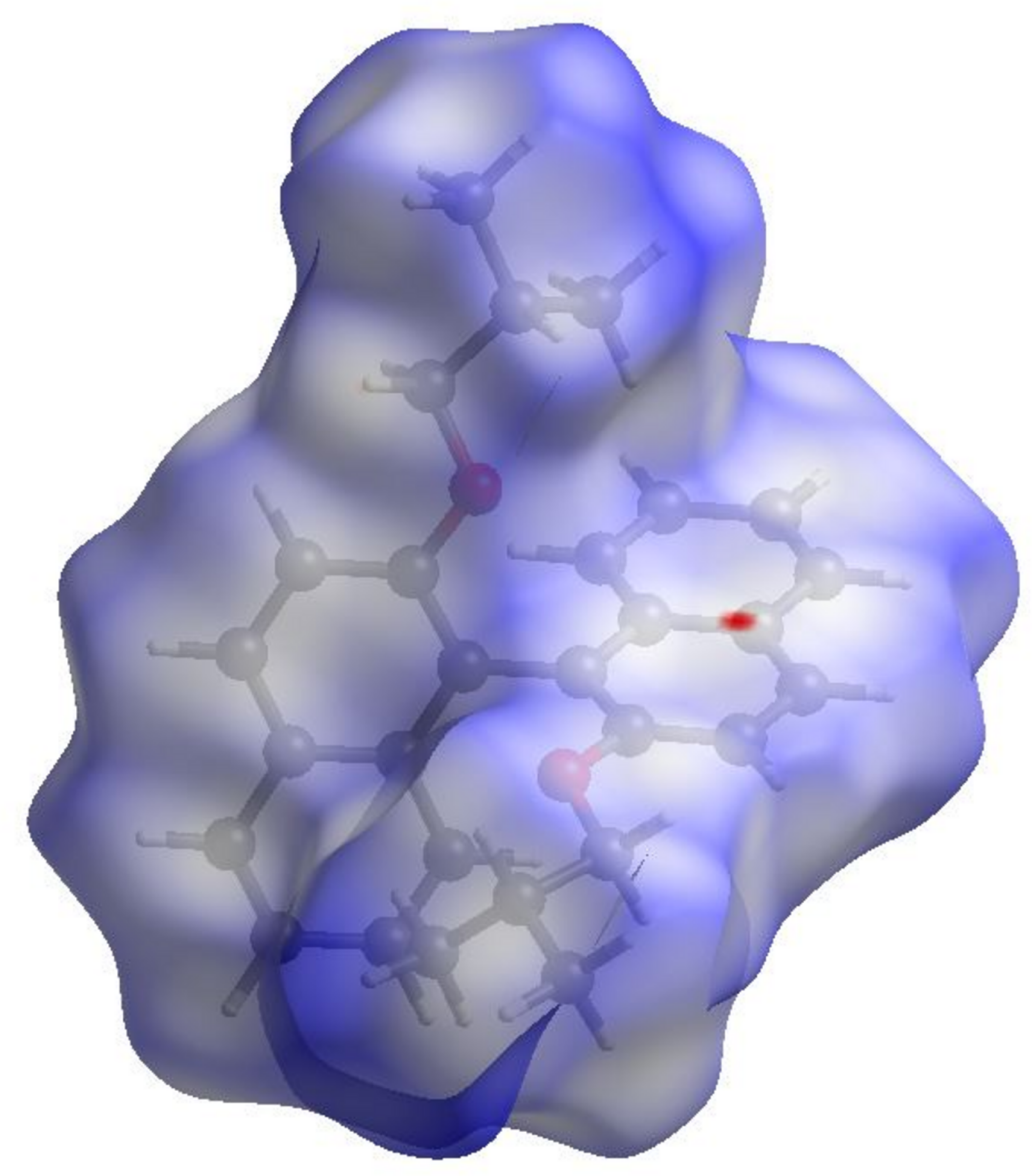
The three-dimensional Hirshfeld surface representation of (I) plotted over *d*_norm._

**Figure 5:**
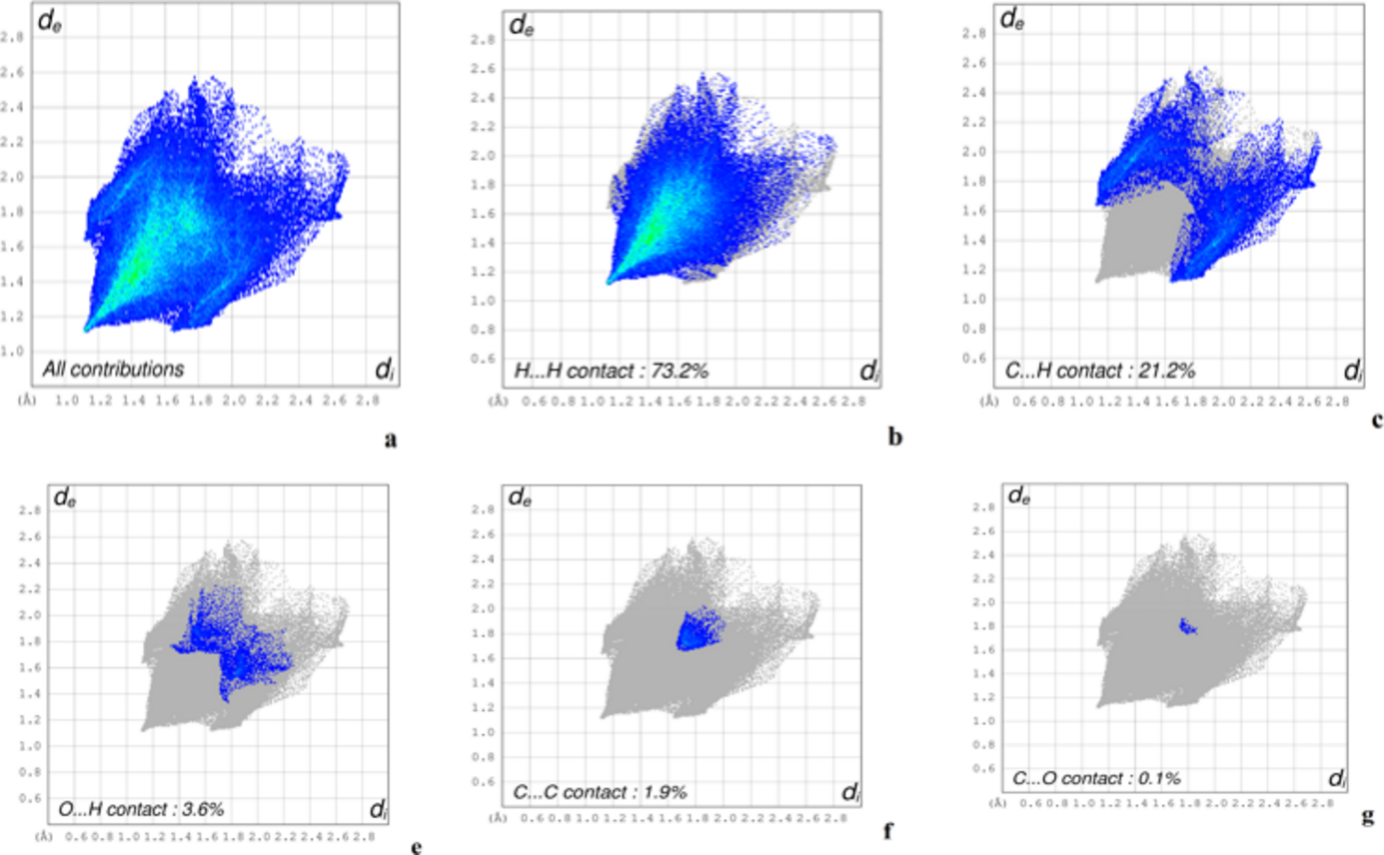
The two-dimensional fingerprint plots of (I) showing (*a*) the overall inter­actions and (*b*)–(*f*) those delineated into H⋯H, C⋯H/H⋯C, O⋯H/H⋯O, C⋯C and O⋯C/C⋯O contacts, respectively.

In order to qu­antify inter­molecular inter­actions revealed by the *PLATON* analysis, Hirshfeld surface (HS) analysis was performed using *CrystalExplorer21.5* (Spackman *et al.*, 2021 ▸). Fig. 4 ▸ shows the three-dimensional HS of (I) mapped over *d*_norm_ on a scale ranging from −0.025 to 1.51 a.u. where the colour highlights the different inter­molecular contacts: blue, white and red regions indicate contacts whose distances are almost equal, longer and shorter, respectively, than the sum of van der Waals radii. Fig. 5 ▸ displays the two-dimensional fingerprint plots of (*d*_i_, *d*_e_) points from all contacts contributing to the HS for all atoms. The H⋯H contacts are the most significant inter­molecular inter­actions and contribute 73.2% whilst C⋯H/H⋯C contacts, which correspond to H⋯π stacking inter­actions, contribute 21.2%. As expected, the H⋯O contacts make a very weak contribution to the crystal packing at 3.6% and the other contacts C⋯C (1.9%) and C⋯O/O⋯C (0.1%) have a negligible contribution.

## 6. Synthesis and crystallization

BINOL was produced according to the method described by McKillop *et al.* (1980 ▸). In a 50 ml flask equipped with a magnetic stirrer, 1 eq. (0.70 mmol, 0.20 g) of BINOL was dissolved in 10 ml of ethanol and 10 eq of sodium hydroxide were added. The mixture was refluxed for 1 h and 21 eq. (14.70 mmol, 1.58 ml) of 1-bromo-2-methyl­propane were added, then heating was maintained for 24 h. Following CCM, at the end of the reaction, several extractions with ethyl acetate were performed and the organic phase was dried with NaSO_4_, then concentrated under reduced pressure. The crude product was purified on a chromatographic silica gel column using hexa­ne/ethyl acetate 90/10 as eluent. A chick yellow powder (60 mg, 21%) was obtained. Single crystals of (I) suitable for X-ray diffraction analysis were grown by slow evaporation from the mixed solvents of hexane and ethyl acetate at room temperature.
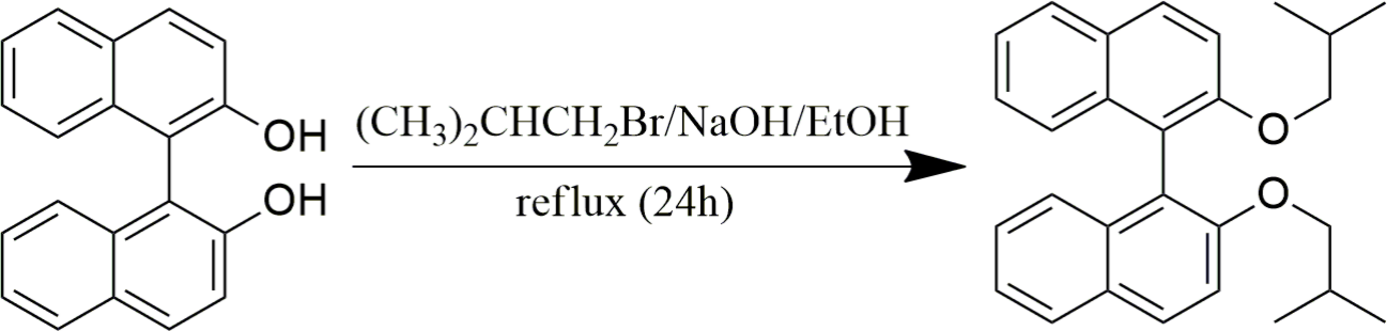


Chick yellow powder, yield = 47%, m.p = 387–389 K. ^1^H NMR (500 MHz, CDCl_3_) δ (ppm) 7.88 (*d*, *J* = 9.0 Hz, 2H, HAr), 7.81 (*dt*, *J* = 8.2, 1.1 Hz, 2H, HAr), 7.36 (*d*, *J* = 9.0 Hz, 2H, HAr), 7.26 (*ddd*, *J* = 8.1, 5.8, 2.1 Hz, 2H, HAr), 7.21–7.13 (*m*, 4H, HAr), 3.67 (*qd*, *J* = 8.8, 6.4 Hz, 4H, –CH_2_–), 1.67 [*dh*, *J* = 13.2, 6.7 Hz, 2H, –CH–(CH_3_)_2_], 0.56 [*d*, *J* = 6.7 Hz, 6H, (CH_3_) –CH–], 0.54 [*d*, *J* = 6.7 Hz, 6H, (CH_3_)–CH–]. ^13^C NMR (126 MHz, CDCl_3_) δ (ppm) 154.66, 134.44, 129.30, 129.09, 127.85, 126.10, 125.65, 123.42, 120.71, 115.62, 76.06, 28.45, 18.98.

## 7. Refinement details

The crystal structure of (I) was refined using *SHELXL* (Sheldrick, 2015*b* ▸) with the standard independent atom model (IAM). Subsequently, this structural model was used as a starting point in a non-spherical refinement procedure. The computational wavefunctions were determined with the *ORCA* program (Neese *et al.*, 2020 ▸) using the DFT method at the PBE0/def2-TZVP level of theory. The non-spherical atomic form factors were calculated using *NoSpherA2* (Kleemiss *et al.*, 2021 ▸). Final refinements were performed with *OLEX2.refine* (Bourhis *et al.*, 2015 ▸). All atoms including H atoms were refined anisotropically. Crystal data, data collection and structure refinement details for the last least-squares refinement are summarized in Table 2 ▸. This process leads to improved precision of the geometrical parameters and more physically realistic C—H separations. For example, the refined C2—O1 bond length obtained here with the non-spherical model is 1.3622 (3) Å compared to 1.3652 (5) Å with IAM and the refined C2—O1—C11 bond angles are 118.315 (17)° (non-spherical) and 118.13 (3)° (IAM). For the background to non-spherical refinement, see Sanjuan-Szklarz *et al.* (2020 ▸) and Jha *et al.* (2023 ▸).

## Supplementary Material

Crystal structure: contains datablock(s) I, global. DOI: 10.1107/S2056989024009101/hb8104sup1.cif

Structure factors: contains datablock(s) I. DOI: 10.1107/S2056989024009101/hb8104Isup2.hkl

CCDC reference: 2384706

Additional supporting information:  crystallographic information; 3D view; checkCIF report

## Figures and Tables

**Table 1:** Hydrogen-bond geometry (Å, °) *Cg*1 and *Cg*2 are the centroids of the C5–C10 and C5′–C10′ rings, respectively.

*D*—H⋯*A*	*D*—H	H⋯*A*	*D*⋯*A*	*D*—H⋯*A*
C13—H13*A*⋯*Cg*2	1.091 (4)	2.998 (5)	4.0338 (4)	158.7 (3)
C13′—H13*E*⋯*Cg*1	1.091 (5)	2.877 (5)	3.8615 (4)	150.1 (3)
C13—H13*C*⋯*Cg*2^i^	1.083 (5)	2.987 (5)	3.6455 (4)	119.6 (3)

Symmetry code: (i) 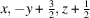.

**Table 2:** Experimental details

Crystal data	
Chemical formula	C_28_H_30_O_2_
*M*_r_	398.55
Crystal system, space group	Monoclinic, *P*2_1_/*c*
Temperature (K)	100
*a*, *b*, *c* (Å)	11.3889 (8), 15.6537 (11), 12.5193 (9)
β (°)	99.920 (2)
*V* (Å^3^)	2198.6 (3)
*Z*	4
Radiation type	Mo *K*α
μ (mm^−1^)	0.07
Crystal size (mm)	0.48 × 0.31 × 0.23

Data collection	
Diffractometer	Bruker D8 Venture
Absorption correction	Multi-scan (*SADABS*; Krause et al., 2015 ▸)
*T*_min_, *T*_max_	0.000, 0.000
No. of measured, independent and observed [*I* ≥ 2u(*I*)] reflections	215936, 17714, 14265
*R*_int_	0.038
(sin θ/λ)_max_ (Å^−1^)	0.989

Refinement	
*R*[*F*^2^ > 2σ(*F*^2^)], *wR*(*F*^2^), *S*	0.023, 0.050, 1.08
No. of reflections	17714
No. of parameters	541
H-atom treatment	All H-atom parameters refined
Δρ_max_, Δρ_min_ (e Å^−3^)	0.20, −0.17

Computer programs: *APEX4* and *SAINT* (Bruker, 2019 ▸), *SHELXT2018/2* (Sheldrick, 2015*a* ▸), *OLEX2.refine* (Bourhis *et al.*, 2015 ▸) and *OLEX2* (Dolomanov *et al.*, 2009 ▸).

## supplementary crystallographic information

### 2,2'-Diisobutoxy-1,1'-binaphthalene Crystal data

**Table:**

C_28_H_30_O_2_	*D*_x_ = 1.204 Mg m^−^^3^
*M**_r_* = 398.55	Melting point: 389 K
Monoclinic, *P*2_1_/*c*	Mo *K*α radiation, λ = 0.71073 Å
*a* = 11.3889 (8) Å	Cell parameters from 17714 reflections
*b* = 15.6537 (11) Å	θ = 3.0–44.7°
*c* = 12.5193 (9) Å	µ = 0.07 mm^−^^1^
β = 99.920 (2)°	*T* = 100 K
*V* = 2198.6 (3) Å^3^	Prism, colourless
*Z* = 4	0.48 × 0.31 × 0.23 mm
*F*(000) = 856.478	

### 2,2'-Diisobutoxy-1,1'-binaphthalene Data collection

**Table:**

Bruker D8 Venture diffractometer	17714 independent reflections
Radiation source: fine-focus sealed tube	14265 reflections with *I*≥ 2u(*I*)
Mirror monochromator	*R*_int_ = 0.038
φ and ω scan	θ_max_ = 44.7°, θ_min_ = 3.0°
Absorption correction: multi-scan (SADABS; Krause *et al.*, 2015)	*h* = −22→22
*T*_min_ = 0.000, *T*_max_ = 0.000	*k* = −30→30
215936 measured reflections	*l* = −24→24

### 2,2'-Diisobutoxy-1,1'-binaphthalene Refinement

**Table:**

Refinement on *F*^2^	Primary atom site location: structure-invariant direct methods
Least-squares matrix: full	Secondary atom site location: structure-invariant direct methods
*R*[*F*^2^ > 2σ(*F*^2^)] = 0.023	Hydrogen site location: difference Fourier map
*wR*(*F*^2^) = 0.050	All H-atom parameters refined
*S* = 1.08	*w* = 1/[σ^2^(*F*_o_^2^) + (0.0137*P*)^2^ + 0.0711*P*] where *P* = (*F*_o_^2^ + 2*F*_c_^2^)/3
17714 reflections	(Δ/σ)_max_ = 0.002
541 parameters	Δρ_max_ = 0.20 e Å^−^^3^
0 restraints	Δρ_min_ = −0.17 e Å^−^^3^
0 constraints	

### 2,2'-Diisobutoxy-1,1'-binaphthalene Fractional atomic coordinates and isotropic or equivalent isotropic displacement parameters (Å^2^)

**Table:**

	*x*	*y*	*z*	*U*_iso_*/*U*_eq_	
O1'	0.776199 (14)	0.498356 (11)	0.341745 (13)	0.01666 (3)	
O1	0.734447 (14)	0.575536 (12)	0.638617 (13)	0.01737 (3)	
C13'	0.59639 (2)	0.44710 (2)	0.15859 (2)	0.02417 (4)	
C12'	0.70148 (2)	0.391771 (15)	0.210094 (18)	0.01879 (4)	
C11'	0.80870 (2)	0.444976 (15)	0.259094 (18)	0.01713 (3)	
C2'	0.857985 (17)	0.555997 (14)	0.390229 (16)	0.01385 (3)	
C1'	0.816020 (16)	0.618843 (13)	0.452436 (15)	0.01271 (3)	
C1	0.686874 (16)	0.621349 (13)	0.458654 (16)	0.01309 (3)	
C2	0.648288 (17)	0.598891 (14)	0.554230 (17)	0.01449 (3)	
C11	0.70253 (2)	0.567336 (16)	0.743665 (18)	0.01806 (4)	
C12	0.81506 (2)	0.547883 (16)	0.824239 (18)	0.01962 (4)	
C13	0.90482 (2)	0.62078 (2)	0.83150 (2)	0.02380 (4)	
C14	0.78109 (3)	0.52831 (2)	0.93444 (2)	0.03137 (6)	
C3	0.525397 (19)	0.598393 (16)	0.56113 (2)	0.01904 (4)	
C4	0.442362 (19)	0.617508 (17)	0.47119 (2)	0.02111 (4)	
C10	0.477288 (18)	0.639919 (15)	0.37163 (2)	0.01831 (4)	
C9	0.601102 (18)	0.643888 (14)	0.366043 (17)	0.01499 (3)	
C14'	0.74018 (3)	0.331031 (18)	0.12705 (2)	0.02435 (5)	
C3'	0.980012 (19)	0.551723 (16)	0.380747 (18)	0.01786 (3)	
C4'	1.059045 (19)	0.609785 (17)	0.43474 (2)	0.01970 (4)	
C10'	1.020820 (18)	0.674390 (15)	0.500151 (18)	0.01737 (4)	
C5'	1.10225 (2)	0.733859 (18)	0.55771 (2)	0.02357 (5)	
C6'	1.06387 (3)	0.796215 (18)	0.62076 (2)	0.02631 (5)	
C7'	0.94151 (3)	0.801770 (17)	0.62857 (2)	0.02385 (5)	
C8'	0.86073 (2)	0.744848 (15)	0.574119 (19)	0.01854 (4)	
C9'	0.897770 (17)	0.679042 (14)	0.508850 (16)	0.01448 (3)	
C8	0.63491 (2)	0.670193 (16)	0.266644 (19)	0.01898 (4)	
C7	0.55041 (2)	0.688558 (18)	0.17723 (2)	0.02395 (5)	
C6	0.42742 (2)	0.681410 (19)	0.18200 (2)	0.02650 (5)	
C5	0.39219 (2)	0.658060 (18)	0.27738 (2)	0.02444 (5)	
H13D	0.6189 (4)	0.4843 (3)	0.0920 (4)	0.0446 (13)	
H13E	0.5691 (4)	0.4921 (3)	0.2162 (4)	0.0384 (12)	
H13F	0.5196 (4)	0.4074 (4)	0.1267 (4)	0.0510 (15)	
H12'	0.6746 (4)	0.3538 (3)	0.2761 (3)	0.0326 (11)	
H11C	0.8815 (4)	0.4033 (3)	0.2933 (4)	0.0337 (11)	
H11D	0.8373 (4)	0.4850 (3)	0.1965 (3)	0.0306 (10)	
H11A	0.6380 (4)	0.5162 (3)	0.7433 (4)	0.0360 (11)	
H11B	0.6621 (4)	0.6265 (3)	0.7650 (3)	0.0355 (11)	
H12	0.8545 (4)	0.4902 (3)	0.7947 (4)	0.0343 (11)	
H13A	0.9295 (4)	0.6347 (3)	0.7529 (4)	0.0387 (12)	
H13B	0.9853 (5)	0.6062 (4)	0.8885 (4)	0.0524 (15)	
H13C	0.8669 (5)	0.6788 (3)	0.8581 (4)	0.0472 (14)	
H14A	0.7414 (5)	0.5840 (4)	0.9646 (4)	0.0519 (15)	
H14B	0.8591 (5)	0.5115 (4)	0.9937 (4)	0.0610 (17)	
H14C	0.7172 (5)	0.4763 (4)	0.9293 (4)	0.0532 (15)	
H3	0.4960 (4)	0.5800 (3)	0.6352 (4)	0.0368 (12)	
H4	0.3491 (4)	0.6135 (3)	0.4759 (4)	0.0402 (12)	
H14D	0.6658 (4)	0.2919 (3)	0.0891 (4)	0.0437 (13)	
H14E	0.8111 (5)	0.2894 (3)	0.1636 (4)	0.0430 (13)	
H14F	0.7714 (5)	0.3666 (3)	0.0630 (4)	0.0430 (13)	
H3'	1.0126 (4)	0.5020 (3)	0.3349 (4)	0.0362 (11)	
H4'	1.1523 (4)	0.6049 (3)	0.4296 (4)	0.0378 (12)	
H5'	1.1953 (4)	0.7284 (3)	0.5514 (4)	0.0402 (12)	
H6'	1.1258 (4)	0.8434 (3)	0.6632 (4)	0.0470 (14)	
H7'	0.9117 (4)	0.8505 (3)	0.6778 (4)	0.0439 (13)	
H8'	0.7674 (4)	0.7488 (3)	0.5805 (4)	0.0316 (11)	
H8	0.7285 (4)	0.6756 (3)	0.2629 (3)	0.0335 (11)	
H7	0.5776 (4)	0.7082 (3)	0.1026 (4)	0.0428 (13)	
H6	0.3615 (4)	0.6941 (3)	0.1094 (4)	0.0448 (13)	
H5	0.2992 (4)	0.6533 (3)	0.2823 (4)	0.0424 (13)	

### 2,2'-Diisobutoxy-1,1'-binaphthalene Atomic displacement parameters (Å^2^)

**Table:**

	*U*^11^	*U*^22^	*U*^33^	*U*^12^	*U*^13^	*U*^23^
O1'	0.01473 (6)	0.01960 (7)	0.01643 (6)	−0.00056 (5)	0.00487 (5)	−0.00336 (5)
O1	0.01438 (6)	0.02456 (8)	0.01393 (6)	0.00018 (5)	0.00453 (4)	0.00063 (5)
C13'	0.02086 (10)	0.03031 (12)	0.02154 (10)	−0.00184 (9)	0.00422 (8)	−0.00185 (9)
C12'	0.02491 (9)	0.01793 (9)	0.01485 (7)	−0.00341 (7)	0.00716 (7)	−0.00102 (6)
C11'	0.01920 (8)	0.01764 (8)	0.01564 (7)	0.00106 (7)	0.00607 (6)	−0.00095 (6)
C2'	0.01112 (6)	0.01761 (8)	0.01319 (6)	0.00082 (5)	0.00311 (5)	0.00077 (6)
C1'	0.01002 (6)	0.01598 (7)	0.01205 (6)	−0.00073 (5)	0.00165 (5)	0.00026 (5)
C1	0.00996 (6)	0.01569 (7)	0.01349 (6)	−0.00009 (5)	0.00166 (5)	−0.00110 (5)
C2	0.01134 (6)	0.01750 (8)	0.01524 (7)	−0.00087 (6)	0.00397 (5)	−0.00182 (6)
C11	0.01926 (8)	0.02077 (9)	0.01563 (7)	−0.00217 (7)	0.00720 (6)	0.00007 (6)
C12	0.02480 (10)	0.02033 (9)	0.01447 (7)	−0.00012 (7)	0.00543 (7)	0.00196 (7)
C13	0.02319 (10)	0.02987 (12)	0.01826 (9)	−0.00493 (9)	0.00332 (8)	0.00032 (8)
C14	0.04086 (16)	0.03786 (16)	0.01659 (9)	−0.00750 (13)	0.00833 (10)	0.00571 (10)
C3	0.01227 (7)	0.02384 (10)	0.02220 (9)	−0.00155 (6)	0.00635 (6)	−0.00351 (7)
C4	0.01030 (7)	0.02574 (10)	0.02732 (10)	−0.00002 (7)	0.00334 (7)	−0.00593 (8)
C10	0.01116 (7)	0.01935 (9)	0.02290 (9)	0.00211 (6)	−0.00134 (6)	−0.00484 (7)
C9	0.01172 (6)	0.01579 (8)	0.01639 (7)	0.00122 (5)	−0.00054 (5)	−0.00140 (6)
C14'	0.03660 (13)	0.01943 (10)	0.01878 (9)	−0.00291 (9)	0.00972 (9)	−0.00368 (8)
C3'	0.01218 (7)	0.02384 (10)	0.01839 (8)	0.00213 (6)	0.00501 (6)	0.00207 (7)
C4'	0.01049 (7)	0.02758 (11)	0.02121 (9)	−0.00055 (7)	0.00324 (6)	0.00521 (8)
C10'	0.01166 (7)	0.02219 (9)	0.01729 (8)	−0.00383 (6)	−0.00025 (6)	0.00516 (7)
C5'	0.01586 (8)	0.02704 (11)	0.02524 (10)	−0.00856 (8)	−0.00369 (7)	0.00664 (8)
C6'	0.02446 (11)	0.02484 (11)	0.02592 (11)	−0.01121 (9)	−0.00610 (8)	0.00256 (9)
C7'	0.02753 (11)	0.02049 (10)	0.02134 (9)	−0.00791 (8)	−0.00194 (8)	−0.00221 (8)
C8'	0.01907 (8)	0.01821 (9)	0.01760 (8)	−0.00435 (7)	0.00107 (6)	−0.00208 (6)
C9'	0.01218 (6)	0.01710 (8)	0.01350 (7)	−0.00279 (6)	0.00037 (5)	0.00179 (6)
C8	0.01749 (8)	0.02077 (9)	0.01702 (8)	0.00143 (7)	−0.00166 (6)	0.00284 (7)
C7	0.02476 (10)	0.02396 (10)	0.01983 (9)	0.00367 (8)	−0.00547 (8)	0.00346 (8)
C6	0.02236 (10)	0.02659 (11)	0.02585 (11)	0.00696 (8)	−0.00914 (8)	−0.00190 (9)
C5	0.01440 (8)	0.02673 (11)	0.02878 (11)	0.00514 (7)	−0.00592 (8)	−0.00621 (9)
H13D	0.043 (3)	0.049 (3)	0.044 (3)	0.013 (3)	0.012 (2)	0.014 (3)
H13E	0.035 (3)	0.044 (3)	0.038 (3)	0.003 (2)	0.009 (2)	−0.004 (2)
H13F	0.035 (3)	0.061 (4)	0.055 (4)	−0.010 (3)	0.000 (3)	−0.014 (3)
H12'	0.046 (3)	0.031 (3)	0.023 (2)	−0.009 (2)	0.012 (2)	−0.0006 (19)
H11C	0.033 (3)	0.035 (3)	0.034 (3)	0.006 (2)	0.006 (2)	−0.005 (2)
H11D	0.034 (3)	0.033 (2)	0.028 (2)	−0.008 (2)	0.012 (2)	−0.003 (2)
H11A	0.038 (3)	0.042 (3)	0.030 (3)	−0.012 (2)	0.011 (2)	0.000 (2)
H11B	0.040 (3)	0.041 (3)	0.027 (2)	0.008 (2)	0.010 (2)	−0.002 (2)
H12	0.046 (3)	0.029 (3)	0.028 (3)	0.006 (2)	0.006 (2)	0.001 (2)
H13A	0.039 (3)	0.049 (3)	0.030 (3)	−0.009 (2)	0.011 (2)	0.003 (2)
H13B	0.041 (3)	0.072 (4)	0.039 (3)	−0.012 (3)	−0.008 (2)	0.012 (3)
H13C	0.047 (3)	0.042 (3)	0.054 (3)	−0.012 (3)	0.014 (3)	−0.011 (3)
H14A	0.063 (4)	0.065 (4)	0.032 (3)	−0.008 (3)	0.021 (3)	−0.002 (3)
H14B	0.061 (4)	0.093 (5)	0.025 (3)	−0.006 (4)	−0.001 (3)	0.018 (3)
H14C	0.069 (4)	0.060 (4)	0.034 (3)	−0.025 (3)	0.016 (3)	0.011 (3)
H3	0.023 (2)	0.053 (3)	0.037 (3)	−0.002 (2)	0.011 (2)	−0.001 (2)
H4	0.022 (2)	0.056 (3)	0.044 (3)	0.004 (2)	0.007 (2)	−0.006 (3)
H14D	0.051 (3)	0.046 (3)	0.037 (3)	−0.014 (3)	0.017 (2)	−0.019 (2)
H14E	0.060 (3)	0.034 (3)	0.037 (3)	0.007 (3)	0.014 (3)	−0.004 (2)
H14F	0.061 (4)	0.041 (3)	0.032 (3)	−0.005 (3)	0.023 (3)	−0.004 (2)
H3'	0.026 (2)	0.040 (3)	0.045 (3)	0.003 (2)	0.014 (2)	−0.002 (2)
H4'	0.023 (2)	0.047 (3)	0.043 (3)	0.000 (2)	0.006 (2)	0.002 (2)
H5'	0.024 (2)	0.042 (3)	0.052 (3)	−0.013 (2)	0.001 (2)	0.003 (3)
H6'	0.033 (3)	0.057 (3)	0.045 (3)	−0.016 (3)	−0.008 (2)	−0.012 (3)
H7'	0.039 (3)	0.043 (3)	0.048 (3)	−0.013 (2)	0.003 (2)	−0.009 (3)
H8'	0.032 (2)	0.027 (2)	0.036 (3)	−0.002 (2)	0.005 (2)	−0.012 (2)
H8	0.031 (2)	0.045 (3)	0.024 (2)	0.004 (2)	0.0031 (19)	0.013 (2)
H7	0.045 (3)	0.048 (3)	0.030 (3)	0.006 (3)	−0.007 (2)	0.011 (2)
H6	0.032 (3)	0.050 (3)	0.046 (3)	0.011 (2)	−0.011 (2)	0.008 (3)
H5	0.026 (3)	0.056 (3)	0.041 (3)	0.003 (2)	−0.005 (2)	−0.006 (3)

### 2,2'-Diisobutoxy-1,1'-binaphthalene Geometric parameters (Å, º)

**Table:**

O1'—C11'	1.4276 (3)	C3—C4	1.3729 (4)
O1'—C2'	1.3621 (3)	C3—H3	1.077 (4)
O1—C2	1.3622 (3)	C4—C10	1.4162 (4)
O1—C11	1.4293 (3)	C4—H4	1.075 (4)
C13'—C12'	1.5272 (4)	C10—C9	1.4252 (3)
C13'—H13D	1.083 (5)	C10—C5	1.4206 (3)
C13'—H13E	1.091 (5)	C9—C8	1.4253 (3)
C13'—H13F	1.091 (5)	C14'—H14D	1.086 (5)
C12'—C11'	1.5176 (3)	C14'—H14E	1.076 (5)
C12'—C14'	1.5287 (4)	C14'—H14F	1.086 (5)
C12'—H12'	1.103 (4)	C3'—C4'	1.3724 (4)
C11'—H11C	1.084 (4)	C3'—H3'	1.071 (4)
C11'—H11D	1.096 (4)	C4'—C10'	1.4162 (4)
C2'—C1'	1.3896 (3)	C4'—H4'	1.078 (4)
C2'—C3'	1.4166 (3)	C10'—C5'	1.4197 (3)
C1'—C1	1.4867 (3)	C10'—C9'	1.4261 (3)
C1'—C9'	1.4238 (3)	C5'—C6'	1.3733 (5)
C1—C2	1.3891 (3)	C5'—H5'	1.080 (4)
C1—C9	1.4258 (3)	C6'—C7'	1.4163 (4)
C2—C3	1.4170 (3)	C6'—H6'	1.093 (4)
C11—C12	1.5190 (4)	C7'—C8'	1.3744 (3)
C11—H11A	1.086 (4)	C7'—H7'	1.073 (5)
C11—H11B	1.088 (4)	C8'—C9'	1.4232 (3)
C12—C13	1.5243 (4)	C8'—H8'	1.081 (4)
C12—C14	1.5268 (4)	C8—C7	1.3750 (3)
C12—H12	1.101 (4)	C8—H8	1.078 (4)
C13—H13A	1.091 (4)	C7—C6	1.4167 (4)
C13—H13B	1.085 (5)	C7—H7	1.078 (5)
C13—H13C	1.083 (5)	C6—C5	1.3732 (5)
C14—H14A	1.079 (6)	C6—H6	1.093 (4)
C14—H14B	1.088 (5)	C5—H5	1.074 (5)
C14—H14C	1.086 (5)		
			
C2'—O1'—C11'	117.900 (17)	H14C—C14—H14B	108.6 (4)
C11—O1—C2	118.315 (17)	C4—C3—C2	119.79 (2)
H13D—C13'—C12'	110.9 (3)	H3—C3—C2	120.7 (2)
H13E—C13'—C12'	112.2 (3)	H3—C3—C4	119.4 (2)
H13E—C13'—H13D	107.1 (4)	C10—C4—C3	121.17 (2)
H13F—C13'—C12'	110.6 (3)	H4—C4—C3	119.3 (3)
H13F—C13'—H13D	107.8 (4)	H4—C4—C10	119.5 (3)
H13F—C13'—H13E	107.9 (4)	C9—C10—C4	118.97 (2)
C11'—C12'—C13'	112.14 (2)	C5—C10—C4	121.71 (2)
C14'—C12'—C13'	111.25 (2)	C5—C10—C9	119.32 (2)
C14'—C12'—C11'	108.06 (2)	C10—C9—C1	119.63 (2)
H12'—C12'—C13'	108.9 (2)	C8—C9—C1	122.086 (19)
H12'—C12'—C11'	107.5 (2)	C8—C9—C10	118.285 (19)
H12'—C12'—C14'	108.9 (2)	H14D—C14'—C12'	110.7 (3)
C12'—C11'—O1'	108.854 (18)	H14E—C14'—C12'	111.7 (2)
H11C—C11'—O1'	110.1 (2)	H14E—C14'—H14D	108.4 (4)
H11C—C11'—C12'	109.6 (2)	H14F—C14'—C12'	110.7 (3)
H11D—C11'—O1'	109.3 (2)	H14F—C14'—H14D	107.2 (4)
H11D—C11'—C12'	110.0 (2)	H14F—C14'—H14E	107.9 (4)
H11D—C11'—H11C	109.0 (3)	C4'—C3'—C2'	119.81 (2)
C1'—C2'—O1'	116.352 (17)	H3'—C3'—C2'	121.0 (2)
C3'—C2'—O1'	122.466 (19)	H3'—C3'—C4'	119.2 (2)
C3'—C2'—C1'	121.164 (19)	C10'—C4'—C3'	121.12 (2)
C1—C1'—C2'	119.137 (17)	H4'—C4'—C3'	119.5 (3)
C9'—C1'—C2'	119.204 (18)	H4'—C4'—C10'	119.3 (3)
C9'—C1'—C1	121.659 (18)	C5'—C10'—C4'	121.51 (2)
C2—C1—C1'	120.070 (17)	C9'—C10'—C4'	118.988 (19)
C9—C1—C1'	120.620 (18)	C9'—C10'—C5'	119.50 (2)
C9—C1—C2	119.275 (18)	C6'—C5'—C10'	120.86 (2)
C1—C2—O1	116.325 (17)	H5'—C5'—C10'	118.3 (3)
C3—C2—O1	122.572 (19)	H5'—C5'—C6'	120.8 (3)
C3—C2—C1	121.080 (19)	C7'—C6'—C5'	119.85 (2)
C12—C11—O1	108.047 (18)	H6'—C6'—C5'	121.1 (3)
H11A—C11—O1	110.0 (2)	H6'—C6'—C7'	119.0 (3)
H11A—C11—C12	110.3 (2)	C8'—C7'—C6'	120.57 (3)
H11B—C11—O1	109.6 (2)	H7'—C7'—C6'	119.8 (2)
H11B—C11—C12	110.6 (2)	H7'—C7'—C8'	119.7 (3)
H11B—C11—H11A	108.3 (3)	C9'—C8'—C7'	120.97 (2)
C13—C12—C11	111.71 (2)	H8'—C8'—C7'	120.6 (2)
C14—C12—C11	108.91 (2)	H8'—C8'—C9'	118.4 (2)
C14—C12—C13	111.60 (2)	C10'—C9'—C1'	119.71 (2)
H12—C12—C11	106.7 (2)	C8'—C9'—C1'	122.050 (19)
H12—C12—C13	109.0 (2)	C8'—C9'—C10'	118.242 (19)
H12—C12—C14	108.7 (2)	C7—C8—C9	120.98 (2)
H13A—C13—C12	111.7 (3)	H8—C8—C9	118.6 (2)
H13B—C13—C12	111.3 (3)	H8—C8—C7	120.4 (2)
H13B—C13—H13A	108.4 (4)	C6—C7—C8	120.51 (3)
H13C—C13—C12	110.4 (3)	H7—C7—C8	120.0 (2)
H13C—C13—H13A	106.7 (4)	H7—C7—C6	119.5 (2)
H13C—C13—H13B	108.1 (4)	C5—C6—C7	119.79 (2)
H14A—C14—C12	109.9 (3)	H6—C6—C7	119.6 (3)
H14B—C14—C12	111.1 (3)	H6—C6—C5	120.6 (3)
H14B—C14—H14A	107.3 (4)	C6—C5—C10	121.04 (2)
H14C—C14—C12	111.6 (3)	H5—C5—C10	118.5 (3)
H14C—C14—H14A	108.0 (4)	H5—C5—C6	120.5 (3)
			
O1'—C11'—C12'—C13'	60.63 (2)	C1—C2—C3—C4	2.26 (3)
O1'—C11'—C12'—C14'	−176.40 (2)	C1—C9—C10—C4	3.02 (2)
O1'—C2'—C1'—C1	−2.31 (2)	C1—C9—C10—C5	−176.67 (2)
O1'—C2'—C1'—C9'	177.507 (17)	C1—C9—C8—C7	177.56 (2)
O1'—C2'—C3'—C4'	−177.60 (2)	C2—C3—C4—C10	−1.58 (3)
O1—C2—C1—C1'	0.17 (2)	C3—C4—C10—C9	−1.05 (3)
O1—C2—C1—C9	178.054 (19)	C3—C4—C10—C5	178.64 (2)
O1—C2—C3—C4	−175.95 (2)	C4—C10—C9—C8	−177.27 (2)
O1—C11—C12—C13	62.99 (2)	C4—C10—C5—C6	178.65 (3)
O1—C11—C12—C14	−173.29 (2)	C10—C9—C8—C7	−2.14 (3)
C2'—C1'—C1—C2	109.31 (2)	C10—C5—C6—C7	−0.70 (3)
C2'—C1'—C1—C9	−68.54 (2)	C9—C8—C7—C6	−0.21 (3)
C2'—C1'—C9'—C10'	0.44 (2)	C3'—C4'—C10'—C5'	178.96 (2)
C2'—C1'—C9'—C8'	−179.856 (19)	C3'—C4'—C10'—C9'	−0.49 (3)
C2'—C3'—C4'—C10'	−0.05 (3)	C4'—C10'—C5'—C6'	179.95 (2)
C1'—C1—C2—C3	−178.14 (2)	C4'—C10'—C9'—C8'	−179.43 (2)
C1'—C1—C9—C10	175.502 (19)	C10'—C5'—C6'—C7'	−0.19 (3)
C1'—C1—C9—C8	−4.19 (2)	C10'—C9'—C8'—C7'	−0.86 (2)
C1'—C9'—C10'—C4'	0.29 (2)	C5'—C6'—C7'—C8'	0.46 (3)
C1'—C9'—C10'—C5'	−179.17 (2)	C6'—C7'—C8'—C9'	0.08 (3)
C1'—C9'—C8'—C7'	179.43 (2)	C8—C7—C6—C5	1.66 (3)

### 2,2'-Diisobutoxy-1,1'-binaphthalene Hydrogen-bond geometry (Å, º)

*Cg*1 and *Cg*2 are the centroids of the C5–C10 and C5'–C10' rings, respectively.

**Table:**

*D*—H···*A*	*D*—H	H···*A*	*D*···*A*	*D*—H···*A*
C13—H13*A*···*Cg*2	1.091 (4)	2.998 (5)	4.0338 (4)	158.7 (3)
C13′—H13*E*···*Cg*1	1.091 (5)	2.877 (5)	3.8615 (4)	150.1 (3)
C13—H13*C*···*Cg*2^i^	1.083 (5)	2.987 (5)	3.6455 (4)	119.6 (3)

Symmetry code: (i) *x*, −*y*+3/2, *z*+1/2.
